# Effects of Olive Oil Phenolic Compounds on Inflammation in the Prevention and Treatment of Coronary Artery Disease

**DOI:** 10.3390/nu9101087

**Published:** 2017-09-30

**Authors:** Priscilla Azambuja Lopes de Souza, Aline Marcadenti, Vera Lúcia Portal

**Affiliations:** 1 Postgraduate Program in Health Sciences: Cardiology, Institute of Cardiology/University Foundation of Cardiology (IC/FUC), Princesa Isabel Avenue, 370, Porto Alegre RS 90620-001, Brazil; pri_azambuja@yahoo.com.br (P.A.L.d.S.); alinemo@ufcspa.edu.br (A.M.); 2 Postgraduate Program in Nutrition Sciences, Federal University of Health Sciences of Porto Alegre (UFCSPA), Sarmento Leite Avenue, 245, Porto Alegre RS 90050-170, Brazil

**Keywords:** olive oil, phenols, inflammation, coronary artery disease

## Abstract

Coronary artery disease (CAD) is responsible for more than 7 million deaths worldwide. In the early stages of the development of atherosclerotic plaques, cardiovascular risk factors stimulate vascular endothelial cells, initiating an inflammatory process, fundamental in the pathogenesis of CAD. The inclusion of potentially cardioprotective foods, such as olive oil, to the diet, may aid in the control of these risk factors, and in the reduction of cytokines and inflammatory markers. The present review aims to address the interaction between phenolic compounds present in olive oil, and inflammation, in the prevention and treatment of CAD. In vitro and in vivo studies suggest that phenolic compounds, such as hydroxytyrosol, tyrosol, and their secoiridoid derivatives, may reduce the expression of adhesion molecules and consequent migration of immune cells, modify the signaling cascade and the transcription network (blocking the signal and expression of the nuclear factor kappa B), inhibit the action of enzymes responsible for the production of eicosanoids, and consequently, decrease circulating levels of inflammatory markers. Daily consumption of olive oil seems to modulate cytokines and inflammatory markers related to CAD in individuals at risk for cardiovascular diseases. However, clinical studies that have evaluated the effects of olive oil and its phenolic compounds on individuals with CAD are still scarce.

## 1. Introduction

Cardiovascular diseases account for 17.7 million deaths every year, and are the leading cause of death worldwide. Of these deaths, it is estimated that 7.4 million are caused by coronary artery disease (CAD) [1]. More than 90% of the events related to CAD—acute myocardial infarction and death—occur in individuals with at least one of the risk factors for CAD [2,3]. Therefore, primary and secondary prevention strategies aim to reduce traditional risk factors (diabetes mellitus, hypertension, dyslipidemia, obesity) and lifestyle-related disorders, such as inadequate diet, smoking, physical inactivity, and abusive use of alcohol [3,4]. In the early stages of development of atherosclerotic plaques, risk factors stimulate vascular endothelial cells to express chemotactic and adhesion molecules, initiating the inflammatory process, fundamental in the pathogenesis of CAD [5,6,7].

In the context of a dietary pattern considered to be healthy, the inclusion of potentially cardioprotective foods, including sources of unsaturated fats and phenolic compounds, such as olive oil, may modulate the concentration of pro-inflammatory cytokines and markers of inflammation [8,9], and assist in the control of modifiable risk factors, such as diabetes mellitus [8], hypertension [10], dyslipidemia [11], and overweight [12,13,14]. Moreover, the consumption of 50 mL/day of extra-virgin olive oil (EVOO) may reduce the chance of developing CAD by 37%, and the incidence of major cardiovascular events by 30% [15].

The concentration of phenolic compounds in EVOO is influenced, among other factors, by the extraction procedure of the oil. EVOO is obtained by mechanical processes, while refined olive oil (ROO) is subjected to both physical and chemical procedures [16,17]. Although ROO presents a similar composition of fatty acids, due to the low phenolic content, it does not bring the same beneficial effects when compared with EVOO [18].

Phenols such as hydroxytyrosol (HT) and derivatives (oleuropein complex and tyrosol) are mainly responsible for the beneficial effects of olive oil in the prevention and progression of atherosclerosis, by improving endothelial function [19], antioxidant effect [20], and the high density lipoprotein (HDL) function [21], reducing the concentration and the atherogenicity of the low density lipoprotein (LDL) [11,22] and inhibiting platelet aggregation [23]. To ensure the cardiovascular benefits of olive oil, the European Food Safety Authority recommends the daily intake of 5 mg of HT or its derivatives, which can be obtained by the daily consumption of at least 20 g of EVOO [24].

Taking into account the potential benefits of olive oil and its phenolic compounds in preventing the mechanisms and the risk factors that may lead to atherosclerosis, and consequently, to the development of cardiovascular disease, the present review aims to address the effects of phenolic compounds present in olive oil on inflammation, both in the prevention and treatment of CAD.

## 2. Inflammatory Process in CAD

Atherosclerosis is a chronic disease initiated by the retention and accumulation of cholesterol-rich lipoproteins, particularly LDL, in the artery wall. Processes such as oxidation of lipoproteins, immunity (innate and adaptive) and inflammation are crucially involved in its pathogenesis [5]. The condition can lead to an acute clinical event caused by the rupture of atherosclerotic plaque and thrombus formation [25,26,27].

Endothelial injury is the event that triggers the formation of atherosclerotic plaques [28]. Risk factors, such as hypertension, dyslipidemia, obesity, diabetes mellitus, smoking, as well as increased oxygen-reactive species, alter the endothelial function and affect the vascular homeostasis by decreasing nitric oxide (NO) synthesis, and altering vessel tone and anti-inflammatory and anticoagulant properties [29,30,31]. As a consequence, an increase in the adhesion of leukocytes and platelets at the vascular injury site, as well as in the permeability of the intimate layer to atherogenic lipoproteins, mainly oxidized LDL, takes place [32].

Retained in the subendothelial space through the binding of apolipoprotein B100 to proteoglycans of the extracellular matrix, LDL particles undergo oxidation [33,34,35]. Endothelial cells activated in response to the inflammatory process produce cytokines (interleukin (IL)-1, IL-6 and tumor necrosis factor-alpha (TNF-α)), chemokines (monocyte chemotactic protein (MCP-1)), adhesion molecules (P and E-selectin, intercellular adhesion molecule-1 (ICAM-1), and vascular cell adhesion molecule-1 (VCAM-1)), and by chemotaxis, cause adhesion and migration of leukocytes (monocytes, B and T lymphocytes) [7,36,37,38,39].

MCP-1 stimulates the migration and infiltration of monocytes into the subendothelial space, through which the macrophage colony-stimulating factor (M-CSF), differentiates into macrophages [40,41]. Macrophages recognize LDLox and begin to express scavenger and toll-like receptors (TLR). TLR regulate the function of macrophages, promoting their activation. Scavenger receptors bind to oxidized LDL and perform phagocytosis, leading to the formation of foam cells, the main components of the fatty striae—the initial atherosclerotic lesions [42,43,44].

The immune response plays an important role in the initiation and development of atherosclerosis. The innate response begins with endothelial and monocyte/macrophage activation, and is followed by the adaptive response, which involves CD4+ T lymphocytes [45].

CD4+ T lymphocytes are among the first cells to be recruited. Within the plaque, they interact with macrophages through the presentation of antigens and differentiate into T helper (Th) cells—Th1 and Th2 lineages [45]. The Th1 phenotype develops in the presence of IL-12 and IL-18 [46,47] (pro-atherogenic response), and is characterized by the secretion of mainly interferon-gamma (INF-γ) [48,49]. In the Th2 subtype, the cytokines IL-4 (which inhibits INF-γ), IL-5, IL-10, and IL-13 are produced, and B cells are activated. The Th2 response is atheroprotective through inhibition of IFN-γ by IL-4 [45,50]. In CAD, there is an imbalance between Th1/Th2, with predominance of pro-inflammatory cytokines [51,52].

In response to the stimulation of IL-6, the liver produces acute phase reagents, such as C-reactive protein (CRP) and fibrinogen [42]. Both are used as biomarkers for diagnostic purposes, as they reflect the level of inflammatory activity [53,54]. CRP can stimulate the expression of both VCAM-1 and ICAM-1 by endothelial cells, and mediate the induction of MCP-1 and the uptake of LDL by macrophages [55,56]. Fibrinogen regulates plasma viscosity while inducing platelet aggregation. In the coagulation cascade, fibrinogen is converted into fibrin by the action of thrombin, and promotes platelet aggregation by binding to the glycoprotein IIb/IIa receptor, consequently increasing the reactivity of platelets [54,57].

One of the main regulators of the inflammatory process at different stages of atherosclerosis is the nuclear factor kappa B (NF-κB). NF-κB is responsible for the regulation of genes coding for chemokines, adhesion molecules, cytokines and proinflammatory acute-phase proteins, cyclooxygenase (COX)-2 enzyme, inducible nitric oxide synthase, apoptosis, and cellular proliferation [58,59]. Another important pathway is mediated by mitogen-activated protein kinases, which regulate cellular processes such as cell growth, proliferation, and differentiation [59]. These two pathways, activated by TLR, increase local inflammatory processes, perpetuating the inflammatory response [60].

COX-1 and COX-2 enzymes are responsible for the production of eicosanoids, prostaglandins, and thromboxanes from arachidonic acid (AA), which is derived from the phospholipid membrane, obtained directly from the diet, or synthesized from linoleic acid [61,62]. COX-1 is expressed in most tissues and plays a role in homeostasis (normal arteries); COX-2, in turn, is expressed in inflammatory cells, and is induced by cytokines such as IL-1, IL-6, and TNF-α (atherosclerotic lesion) [63]. Prostaglandins (PG) act in the recruitment of leukocytes and infiltration of immune cells into the inflammatory site. PGE2 is an important mediator of the inflammatory response, inducing the expression and activity of matrix metalloproteinase-9 (MMP-9) in macrophages [64]. MMP-9 plays a role in angiogenesis and in the formation and vulnerability of the atherosclerotic plaque [65]. Thromboxanes (TX) A2 and B2 (TXA2 and TXB2) increase platelet aggregation and potentiate thrombus formation [66]. Another eicosanoid from AA, through lipoxygenases, is the leukotriene (LT) B4 (LTB4), which has a chemotactic effect on neutrophils, directing the cells to the atherosclerotic lesion [67,68].

## 3. Olive Oil: Classification and Composition

Olive oil is the oil obtained solely from the olive tree fruit (*Olea europaea* L., *Oleacea* family), excluding the use of solvents, re-esterification processes, and mixture with any other types of vegetable oils. Virgin olive oil (VOO) is obtained exclusively by mechanical or other physical means under conditions that do not alter the oil, and is not subjected to any treatment other than washing, decantation, centrifugation, and filtration [16,69].

The virgin olive oils are classified into EVOO, virgin (fine), and lampante, according to the degree of acidity (ratio of free fatty acids to total oleic acid): ≤0.8%, ≤2%, and >2%, respectively. EVOO also differs from fine oil in quality: although both are obtained by physical means, EVOO has superior physicochemical and sensory properties [70].

All virgin olive oils are composed of two fractions: saponifiable and unsaponifiable. The saponifiable fraction (larger components) represents approximately 98% of the oil composition [71], and the oleic monounsaturated fatty acid comprises 55–83% of that fraction. The virgin olive oils also have significant concentrations of polyunsaturated fatty acids (linoleic fatty acid: 3.5–21%) and saturated fatty acids (palmitic fatty acid: 7.5–20%, stearic fatty acid: 0.5–5%) [16].

The unsaponifiable fraction (minor components) constitutes 1–2% of the total content of the virgin oils, and includes more than 230 compounds: (1) sterols (e.g., β-sitosterol); (2) hydrocarbons (e.g., squalene and carotenoids (β-carotene and lycopene)); (3) volatile compounds; (4) triterpenic and aliphatic alcohols; (5) pigments (e.g., chlorophyll); and (6) phenolic compounds [72,73].

### Phenolic Compounds of Olive Oil

Phenolic compounds are secondary plant metabolites synthesized during normal development or in stressful situations [74]. In virgin olive oils, the synthesis of these compounds occurs when the olive fruits are crushed during the industrial process to obtain the olive oil. Thus, the presence of phenolic compounds is directly related to glycosides initially present in the fruit tissue, and the activity of hydrolytic and oxidative enzymes [75]. In terms of chemical structure, they have at least one hydroxyl attached to an aromatic ring [74].

According to their characteristics, phenolic compounds are classified into lipophilic (α, β, and γ-tocopherols and tocotrienols) [76,77,78] or hydrophilic. Among the lipophilic phenolic compounds present in virgin olive oils, α-tocopherol is the most relevant (>90% of tocopherols) [79], with a mean concentration of 150.7 mg/kg [80], and reaching levels of up to 400 mg/kg [77,79]. At least 36 hydrophilic phenolic compounds have been identified in olive oil and grouped into six categories according to their chemical structure [81] (Table 1).

The phenolic compounds are mainly responsible for the organoleptic characteristics (aroma and flavor) [82,83] and oxidative stability of the olive oil [20,84]. Several factors influence their concentration: plant variety [77,84,85], environmental factors [86,87], olive storage and maturation conditions [88,89], oil extraction conditions [90,91] and commercial storage of the final product [92]. The mean phenolic content in EVOO is 551.4 mg/kg [93,94] (ranging from 50–800 mg/kg) [95]; in fine oil, it is 206.7 mg/kg [94], and ROO has the lowest indices, 198–62.0 mg/kg [17,94]. The phenolic content of EVOO has a wide variation. Montano et al., evaluated eight varieties of plants grown at extreme altitudes, and found that Cornicabra presented the highest concentrations (mean of 632.6 mg/kg) while Arbequina had the lowest values (200.2 mg/kg) [84]. Baiano et al., evaluated the effect of planting location on the content of phenolic compounds and found significant variations, from 195.2 to 32.3 mg/kg for the same type of crop [96].

HT (3,4-DHPEA) and tyrosol (*p*-HPEA), and especially its secoiridoid derivatives—the dialdehydic forms of the decarboxymethyl elenolic acid linked to hydroxytyrosol (oleacein: 3,4-DHPEA-EDA) and tyrosol (oleocanthal: *p*-HPEA-EDA), aglycones of oleuropein (3,4-DHPEA-EA) and ligstroside (*p*-HPEA-EA)—are the most abundant phenolic compounds in olive oil (90% of the total phenolic content) [81,97]. Oleuropein may give origin to oleuropein aglycone, hydroxytyrosol, and elenolic acid by hydrolysis. This process, which occurs during the maturation of the olive fruit and extraction and storage of the oil, is in part responsible for the variety and complexity of EVOO flavors [72]. In addition to dryiridoids, lignans are also present in high concentrations, mainly (+)-1-acetoxypinoresinol and (+)-pinoresinol [98].

The levels of phenolic acids, flavonoids and hydroxy-isocromans are relatively low in virgin olive oil. Phenolic acids were the first compounds identified in the olive oil; at least 14 have already been described and are generally present in amounts of less than 1 mg/kg [99,100,101]. Luteolin and apigenin are the two flavonoids found in the highest concentration in olive oil; however, this is lower than that of other phenolic compounds [102]. Bianco et al., identified two hydroxy-isocromans [1-phenyl-6,7-dihydroxy-isochroman and 1-(3′-methoxy-4′-hydroxy)-6,7-dihydroxy-isochroman] in commercial virgins olive oils formed from the HT reaction with benzaldehyde and vanillin, respectively [103]. The average concentrations of the phenolic compounds found in the different types of olive oil are presented in Table 1.

After ingestion, the phenolic compounds are metabolized through two phases: hydrolysis (phase 1) that occurs in the stomach and small intestine (where most are promptly absorbed); and conjugation (phase 2), in the small intestine and mainly in the liver—this process basically involves methylation, sulfation, and glucuronidation [104,105,106,107,108]. Oleuropein, specifically, does not follow the same route, as it is degraded by the colon microbiota to HT, which can then be absorbed [106].

In humans, the absorption of phenolic compounds of virgin oils, especially HT and tyrosol, is dose-dependent, and these compounds are excreted in the urine in the conjugated form [109]. Vissers et al., studied healthy subjects (one group submitted to ileostomy and another with intact colon) and estimated that the absorption rate would be at least 55–66% of the dose of ingested olive oil [110]. The absorption of HT also depends on the food matrix: higher percentages of urinary excretion were observed after ingestion of this phenolic compound as a natural component of virgin olive oils (42% of ingested HT), compared to ROO (23%) or to yogurt (5.8%) [111].

## 4. Phenolic Compounds of Olive Oil and Inflammation: In Vitro and Animal Model Studies

Evidence has shown that regular consumption of foods rich in phenolic compounds may decrease the risk for the development of chronic diseases [112,113], mainly due to their ability to modulate low-grade inflammation [114]. The mechanisms by which these compounds may exert an anti-inflammatory effect, specifically on cardiovascular diseases, involves: (1) antioxidant activity; (2) modification of the signaling cascade and transcription network (blocking the signaling and expression of nuclear factor kappa B); (3) decrease of the adhesion of immune cells (T lymphocytes and monocytes) to the endothelium; and (4) improvement of endothelial dysfunction [114]. Due to the complex chemical composition of the oil, particularly the EVOO, we tried to elucidate which phenols would be more involved in these mechanisms.

In human umbilical vein endothelial cells cultured in vitro and stimulated by lipopolysaccharides (LPS) or cytokines (TNF-α, IL-1β), HT inhibited the endothelial activation and expression of VCAM-1 and ICAM-1 [115,116]. Elenolic acid and tyrosol were also tested, but did not show the same results in reducing VCAM-1 expression [115]. Besides the HT, its phase II metabolites, biosynthesized by intestinal Caco-2 cells (hydroxytyrosol sulfate, hydroxytyrosol 4′-glicuronide, hydroxytyrosol 3′-glicuronide) were also effective in reducing the biomarkers of endothelial dysfunction (MCP-1, E and P- Selectin, ICAM-1, and VCAM-1) [117].

At nutrient-relevant concentrations (<10 μM), close to those found in human fluids following EVOO intake [118], HT inhibited the production of ON and PGE2, but had no effect on the expression of inducible nitric oxide synthase, TNF-α or IL -1β, in granulocytes and monocytes [119]. In peripheral blood mononuclear cells, HT culture reduced MMP-9 concentrations, and inhibited PGE2 production and COX-2 expression, without affecting COX-1 [120]. In endothelial cell culture, HT and oleuropein phenols reduced the inflammatory process in angiogenesis through the inhibition of COX-2 and MMP-9 [121], suggesting that the mechanism of action of HT on the inflammatory process is similar to that of nonsteroidal anti-inflammatory drugs (NSAIDs) [122,123] (inhibition of the COX enzyme results in reduced synthesis of eicosanoids (PG and TX) from AA).

Rosignoli et al., performed an in vitro experiment with the objective of evaluating the effects of different olive oil polyphenols on the modulation of inflammatory mediators in human monocytes. The cells were treated for 24 h with 100 μM HT (3,4-DHPEA), tyrosol (*p*-HPEA) and their secoiridoid derivatives (3,4-DHPEA and *p*-HPEA bound to the dialdehydic form of elenolic acids: 3,4- DHPEA-EDA (oleacein) and *p*-HPEA-EDA (oleochantal), respectively). The evaluated compounds significantly inhibited the production of superoxide anions (O_2_^−^) by 40% (3,4-DHPEA), 9% (*p*-HPEA), 25% (3,4-DHPEA- EDA), and 36% (*p*-HPEA-EDA). HT significantly reduced COX-2 expression (mRNA and protein level) and release of PGE2, the latter being dose-dependent. HT also increased the production of TNF-α by monocytes. COX-2 mRNA was also inhibited by secoiridoid derivatives [123]. 

Tyrosol and hydroxyl-isocroman compounds also have an effect on AA. In macrophage culture (RAW 264.7) stimulated by phorbol-12-myristate-13-acetate esters, tyrosol (≥100 μM) inhibited the release of AA and synthesis of metabolites (PGE2 and LTB4) induced by exogenous oxygen-reactive species. This further reduced the release of NO induced by phorbol-12-myristate-13-acetate stimulus [124]. 1-Phenyl-6,7-dihydroxy-isochroman significantly inhibited the production of TXA2 and PGE2, and of TNF-α in LPS-primed human monocytes; this action was mediated by the suppression of NF-κB activation, leading to a decrease in COX-2 synthesis [125].

In vitro results on AA have also been observed in vivo [126,127]. In healthy subjects, consumption of a meal (150 g of tomatoes) with EVOO (607 mg/kg phenolic content, 300 mg/kg of HT derivatives) reduced the inflammatory markers TXB2 and LTB4 after 2 and 6 h [127].

Oleocanthal is another phenol with anti-inflammatory effects similar to those of NSAIDs. Oleocanthal was able to induce dose-dependent inhibition of COX-1 and COX-2 inflammatory enzymes in vitro, and had higher potential at equimolar concentrations when compared with ibuprofen; 25 mM of oleocanthal inhibited the COX enzyme activity by 41–57% while 25 mM ibuprofen inhibited it by 13–18% [128,129].

In addition to inhibiting the expression of endothelial adhesion molecules (VCAM-1) [115], oleuropein may reduce the inflammatory response by inhibiting TLR and the signaling of mitogen-activated protein kinases in a zebrafish model [130]. Oleuropein administration inhibited proliferation of vascular smooth muscle cells in vitro [131].

Male Sprague Dawly rats were allocated into five groups: (1) the sham group previously treated with vehicle; (2) the acute myocardial infarction group previously treated with vehicle (1 mL of distilled water/day); (3) three acute myocardial infarction groups that received different concentrations of oleuropein (10, 20, and 30 mg/kg) for 7 days before acute myocardial infarction. The groups receiving previous treatment with oleuropein (20 and 30 mg/kg) had lower IL-1β and TNF-α values, when compared to the group with acute myocardial infarction that received only the vehicle [132].

Wister rats were fed, for 9 weeks, a high cholesterol diet or high cholesterol diet supplemented with different types of oils: (1) sunflower oil (SFO); (2) SFO enriched with EVOO phenolic compounds (302 mg/kg) (SFO+); (3) SFO rich in monounsaturated fatty acid (HSFO); (4) SFO rich in monounsaturated fatty acid enriched with EVOO phenolic compounds (341 mg/kg) (HSFO+); (5) EVOO rich in phenolic compounds (168 mg/kg) (EVOO); and (6) EVOO poor in phenolic compounds (28 mg/kg) (EVOO−). In the groups receiving EVOO, EVOO (−), HSFO and HSFO (+), there was attenuation of E-selectin levels in the aorta of the animals. In the EVOO and EVOO (−) groups, the concentration of VCAM-1 (in the aorta) was lower than in those who consumed the high cholesterol diet alone [133].

Hyperhomocysteinemia has been associated with a high risk of cardiovascular disease because it increases vascular endothelial adhesiveness [134]. Phenolic compounds, such as tyrosol and *p*-coumaric acid, may decrease homocysteine-induced cell adhesion and ICAM-1 expression; however, they do not reduce the expression of ICAM-1 induced by TNF-α, demonstrating the potential selective effect of these compounds [135].

Recent studies have suggested the importance of the concomitant presence of non-alcoholic fatty liver disease and systemic inflammation (elevated CRP) in the development of atherosclerosis [136]. The effects of HT were investigated using a high-fat diet in an animal model of insulin resistance and non-alcoholic fatty liver disease. The rats were divided into three groups: (1) control diet (10.5% of lipids); (2) high fat diet (58% lipids); (3) high-fat diet + HT (10 mg/kg/day). After 6 weeks, HT attenuated, significantly, the metabolic impairment induced by the high-fat diet. It had also reduced hepatic inflammation and nitrosative−oxidative stress through decreased protein nitrosylation, lipid peroxidation, and production of oxygen-reactive species [137].

## 5. Studies on Olive Oil Phenolic Compounds and Inflammation in Individuals at Risk for CAD

Studies on primary prevention have demonstrated the association between consumption of EVOO, naturally rich in phenolic compounds, and reduced risk of major cardiovascular events in patients at high risk for developing cardiovascular diseases [15,138]. Such an effect may be mediated by the control of modifiable risk factors and potential anti-inflammatory mechanisms of olive oil phenols [9].

Cardiovascular risk factors, such as hypertension, dyslipidemia, diabetes mellitus, and smoking, cause endothelial dysfunction, contributing to the onset of the inflammatory process in atherosclerosis [5]. Obesity and metabolic syndrome are characterized by a chronic and low-grade inflammatory state, increasing the contribution of inflammation to the genesis and evolution of CAD [139,140]. Thus, nutritional strategies and interventions that minimize the inflammatory process in individuals at high cardiovascular risk would help in the primary prevention of CAD.

Studies have shown the anti-inflammatory effects of virgin olive oils supplementation at different stages of development of atherosclerosis. In individuals with endothelial dysfunction, Widmer et al. evaluated the effects of VOO (340 mg/kg total polyphenols) and VOO enriched with epigallocatechin 3-gallate (EGCG), a catechin naturally found in green tea (VOO + EGCG: 300 mg/kg total polyphenols + 280 mg/Kg EGCG) on inflammatory mediators. The main difference in phenolic composition between the two oils was the secoiridoid content (VOO 61% vs. VOO + EGCG 48%), whereas the lignan content was similar (VOO 33% vs. VOO + EGCG 37%). The authors did not find differences between the VOO and VOO + EGCG groups, but concluded that the supplementation of olive oil to the usual diet of the participants for 4 months had a positive effect on the reduction of cell adhesion molecules (sICAM-1), platelets, monocytes, and lymphocytes involved in the inflammatory process [141].

In the PREDIMED (*PREvención con DIeta MEDiterránea*), study, the Mediterranean diet (MeDiet) supplemented with EVOO (1 liter/week), compared to the control group (low-fat diet), was able to significantly reduce proinflammatory cytokines (IL6) (P-selectin, sVCAM and sICAM) in subjects at high cardiovascular risk (type 2 diabetes mellitus alone or ≥3 other risk factors: hypertension, HDL ≤ 40 mg/dl, LDL ≥ 160 mg/dl, overweight or obesity, smoking, family history of premature CAD), at short- (3 months) and long-term (1 year) follow-up [9,142]. The same study group demonstrated the effects of MeDiet supplementation with EVOO on plasma concentrations of inflammatory molecules and atherosclerotic plaque stability. Participants were evaluated at three time points: at baseline, after 3 years, and after 5 years. In contrast to the control group, there were significant reductions in IL-6, IL-8, MCP-1, MIP-1β, IL-1β, IL-5, IL-7, IL-12p70, IL-18, TNF-α, and IFN -γ at 3 and 5 years, compared to the baseline [143].

Two randomized crossover clinical trials evaluated the anti-inflammatory effects of VOO in patients with mild dyslipidemia (no drug treatment). In the VOLOS study (Virgin Olive Oil Study), participants underwent two interventions for 7 weeks each, 40 mL/day of EVOO containing 166 mg/L of HT (free and esterified in oleuropein), and ROO with only 2 mg/L. The predominant fatty acids in the two samples were oleic (70.9% EVOO vs. 72.7% ROO), palmitic (11.5% vs. 10.7%), and linoleic (8.5% vs. 7.6%). The results demonstrated a 20% reduction in serum TXB2 concentration only in the EVOO group containing HT [127]. The second trial compared MeDiet supplemented with three different dietary sources of fats (35–50 g VOO, 40–65 g nuts, and 50–75 g almonds), accounting for 40% of the dietary lipid content (20% of the total energy value). Interventions were performed for 4 weeks, and although VOO contained the highest total polyphenol content (343 mg/kg vs. 13 mg/kg of walnuts and 11 mg/kg of almonds), there was no significant difference in serum levels of CRP and adhesion molecules (sVCAM-1 and sICAM-1) after follow-up [144]. 

Another short-term intervention (4 weeks), with patients with type 2 diabetes mellitus (without insulin therapy) and overweight, compared the intake of 25 mL/day of EVOO rich in phenolic compounds (577 mg/kg, mainly HT) and ROO (washout period), and did not show differences between the groups (CRP, IL-6, and TNF-α) [145]. On the other hand, women with normal-high blood pressure (systolic blood pressure [SBP] 120–139 mmHg and/or diastolic blood pressure [DBP] 80–89 mmHg) or at stage 1 of essential hypertension (SBP 140–159 mmHg and/or DBP 90–99 mmHg) had reduced CRP concentrations (−1.9 ± 1.3 mg/L; *p* < 0.001) and asymmetric dimethylarginine (−0.09 ± 0.01 μmol/L; *p* < 0.01) by MeDiet supplemented with 60 mL of VOO (564 mg/kg total polyphenols; 30 mg/day total polyphenols) compared to MeDiet with ROO (polyphenol-free) after 8 weeks [19].

A high-fat meal, in addition to promoting postprandial hypertriglyceridemia, stimulates the intestinal absorption of endotoxins such as LPS. This endotoxin is able to bind TLR4, which in turn triggers various signaling pathways, including NF-κB, leading to transcription of genes related to the inflammatory response [146,147]. With the objective of investigating the mechanisms by which the VOO polyphenols reduce the postprandial inflammatory response, Camargo et al., administered a VOO-rich meal with different concentrations of phenolic compounds (high 398 mg/kg, intermediate 149 mg/kg, and low 70 mg/kg) to subjects with metabolic syndrome. After 4 h, high concentrations of VOO inhibited NF-κB and decreased the expression of IL-1β (vs. intermediate), and IL-6 (vs. low/intermediate). The VOO-rich meal with low phenol concentration promoted increased serum levels of IL-6, as well as the protein NF-κB subunit p65, TLR4, and postprandial LPS. These results suggest that the ingestion of phenolic VOO reduces postprandial inflammation mainly by decreasing the activation of NF-κB, secondary to the reduction of LPS absorption [148].

Obese subjects received a breakfast containing milk and muffins made with different oils: (1) VOO—containing 400 mg/kg of antioxidant phenols (monounsaturated fatty acid 70.5%, polyunsaturated fatty acids 11.1%, saturated fatty acids 18.4%); (2) SFO (monounsaturated fatty acid 34.3%, polyunsaturated fatty acids 58.3%, saturated fatty acids 7.3%); (3) SOD—mixture of oils (30% SFO + 70% canola oil) + 2 mg of dimethylpolysiloxane (artificial antioxidant) (monounsaturated fatty acid 71.8%, polyunsaturated fatty acids 18%, saturated fatty acids 12.2%); (4) SOP—mixture of oils (30% SFO + 70% canola oil) + VOO-phenol compounds (400 mg/kg) extracted from the residue of olive oil production alperujo (monounsaturated fatty acid 76.7%, polyunsaturated fatty acids 17.6%, saturated fatty acids 5.8%). The oils were previously subjected to 20 heating cycles. Interventions with VOO and SOP reduced NF-κB activation, increased NF-κB alpha inhibitor, and reduced plasma LPS concentration (2 h). The results suggest that oils rich in phenolic compounds, both natural (VOO) and artificial (SOP), are capable of modulating postprandial inflammation [149].

In addition to the postprandial anti-inflammatory effect of VOO, the ingestion of a EVOO-rich meal (72% of the caloric intake) (1125 mg/kg total polyphenols and 350 mg/kg tocopherols) resulted in lower elevations of adhesion molecules (ICAM-1 and VCAM-1) in hypertriglyceridemic men, and in healthy subjects after 8 h, and compared to a high ROO breakfast [150].

Table 2 shows the main randomized clinical trials that evaluated the effect of different concentrations of olive oil phenolic compounds on inflammation markers in patients with cardiovascular risk.

## 6. Anti-Inflammatory Effects of Olive Oil Phenolic Compounds in Patients with CAD

The anti-inflammatory effects of VOO phenolic compounds have been extensively investigated in vitro, in animal models and in clinical trials involving subjects at risk for cardiovascular diseases. However, few studies have tested its effects specifically in CAD patients. Fitó et al. evaluated the effect of daily supplementation of 50 mL/day of VOO and ROO in patients with stable CAD for two periods of 3 weeks. The oils used in the study had similar monounsaturated fatty acid content and differed mainly in phenolic content (161 vs. 14.7 mg/kg total; 0.15 vs. 0 mg of β-carotene; 8.73 vs. 5.99 mg of α-tocopherol; 6.53 vs. 0.62 mg of caffeic acid equivalents; respectively). Serum concentrations of sICAM-1, sVCAM-1, CRP, and IL-6 were evaluated. The effects on proinflammatory cytokines (IL-6) and inflammation markers (CRP) were observed only in the VOO group, with a reduction of 0.166mg/dL (95% CI −0.261–0.071; *p* = 0.002) and 0.063 mg/dL (95% CI −0.119–0.007, *p* = 0.024), respectively [18] (Table 2).

**Figure 1:**
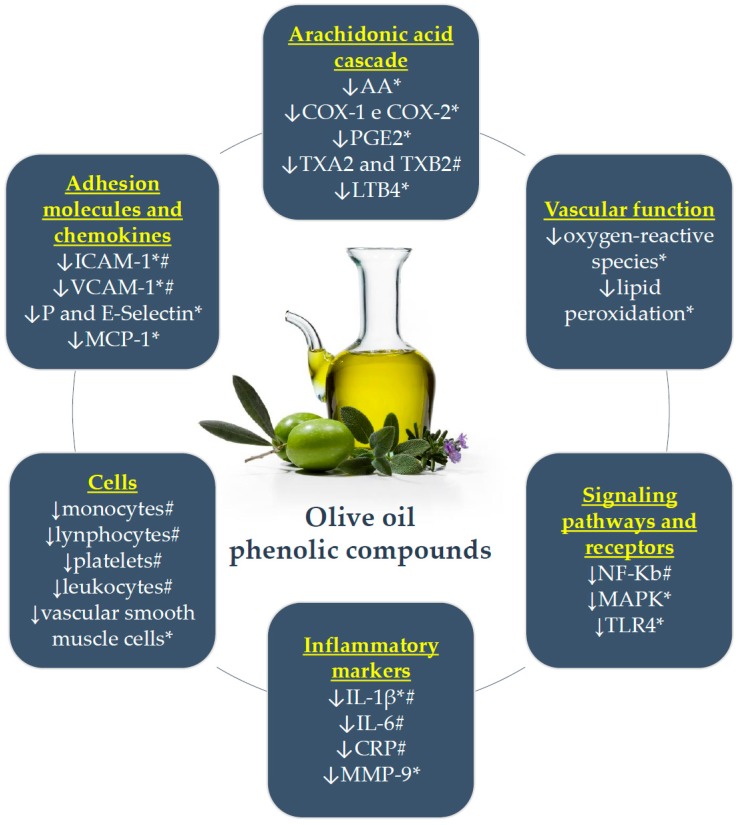
Main anti-inflammatory effects of olive oil phenolic compounds. *: in vitro or animal model; #: individuals at risk for CAD; ↓: decreases or inhibits; AA: arachidonic acid; COX: cyclooxygenase; PGE2: prostaglandins E2; TX: thromboxane; LTB4: leukotriene B4; NF-κB: nuclear factor kappa B; MAPK: mitogen-activated protein kinases; TLR: toll-like receptor; IL: interleukin; CRP: C-reactive protein; MMP-9: matrix metalloproteinase-9; ICAM-1: intercellular adhesion molecule-1; VCAM-1: vascular cell adhesion molecule-1; MCP-1: chemotactic monocyte protein-1.

The main anti-inflammatory effects of olive oil phenolic compounds are briefly presented in Figure 1. 

## 7. Conclusions

The consumption of VOOs rich in phenolic compounds seems to favorably modulate inflammation, which contributes to the development and progression of CAD. In vitro and animal model studies have suggested mechanisms of action of these compounds in inflammatory activity at the cardiovascular level, including effects on the arachidonic acid cascade and on signaling pathways and receptors, improvement of vascular function, and reduction of adhesion molecules and chemokines. Moreover, these studies allow us to evaluate the isolated effects of VOO phenolic compounds. 

Clinical trials conducted in individuals at risk for the development of cardiovascular diseases show positive effects of daily intake of different amounts of olive oil on inflammatory markers. The main findings of the randomized clinical trials included in this review reinforce the results found in in vitro and animal models. In humans, these effects were observed at the cell level (immune cells) and in inflammatory markers. A limitation for the discussion of the results is the great variation in the phenolic content of different types of VOOs. Furthermore, dietary supplementation with olive oil is associated with changes in dietary patterns as a whole, which may improve the inflammatory profile of patients at risk for CAD. It is also important to consider that dietary patterns, like MeDiet, include other sources of phenolic compounds.

As we have seen, the effects of olive oil and/or its phenolic compounds specifically on individuals with established CAD are still scarce. In this sense, more clinical trials, preferably long-term studies, are necessary to evaluate and confirm the beneficial effects of the phenolic compounds present in the olive oil on the inflammatory process, both in the prevention and treatment of CAD.

## Figures and Tables

**Table 1:** Classification of the main hydrophilic phenolic compounds found in virgin olive oils and their average concentration in different types of olive oil.

Chemical Structure	Components	ROO mg/kg * (Mean ± SD)	Virgin (Fine) mg/kg * (Mean ± SD)	EVOO mg/kg * (Mean ± SD)
**Phenolic acids**	benzoic	-	-	-
	gallic	-	-	-
	*p-*hydroxybenzoic	-	0.37 ± 0.37	-
	protocatechuic	-	1.47 ± 0.56	-
	syringic	-	0.81 ± 1.17	0.25 ± 0.25
	vanillic	-	1.22 ± 2.04	0.64 ± 0.50
	caffeic	-	0.21 ± 0.63	0.19 ± 0.45
	cinnamic	-	-	0.17 ± 0.14
	o-coumaric	-	-	-
	*p*-coumaric	-	0.24 ± 0.81	0.92 ± 1.03
	ferulic	-	0.19 ± 0.50	0.19 ± 0.19
	sinapic	-	-	-
**Phenolic alcohols**	hydroxytyrosol (3,4-DHPEA)	6.77 ± 8.26	3.53 ± 10.19	7.7 2 ± 8.81
	tyrosol (*p*-HPEA)	4.11 ± 2.24	5.34 ± 6.98	11.32 ± 8.53
**Secoiridoids**	oleuropein	-	-	1.65 ± 1.85
	oleuropein aglycone	125.40 ± 41.80	120.57 ± 125.53	36.63 ± 24.34
	ligstroside aglycone	59.93 ± 18.58	82.01 ± 67.78	17.44 ± 18.13
	monoaldehydic form of oleuropein aglycone (3,4-DHPEA-EA)	10.90 ± 0.00	95.00 ± 116.01	72.20 ± 64.00
	monoaldehydic form of ligstroside aglycone (*p*-HPEA-EA)	15.20 ± 0.00	69.05 ± 69.00	38.04 ± 17.23
	dialdehydic form of decarboxymethyl elenolic acid linked to hydroxytyrosol (oleacein: 3,4-DHPEA-EDA)	57.37 ± 27.04	77.83 ± 256.09	251.60 ± 263.24
	dialdehydic form of decarboxymethyl elenolic acid linked to tyrosol (oleocanthal: *p*-HPEA-EDA)	38.95 ± 9.29	71.47 ± 61.85	142.77 ± 73.17
**Flavonoids**	flavones			
	luteolin	1.17 ± 0.72	1.29 ± 1.93	3.60 ± 2.32
	apigenin	0.30 ± 0.17	0.97 ± 0.71	11.68 ± 12.78
	flavanonol			
	taxifolin	-	-	-
**Lignans**	(+)-1-acetoxypinoresinol	7.52 ± 9.10	4.43 ± 21.28	6.63 ± 10.78
	(+)-pinoresinol	24.05 ± 10.02	23.71 ± 17.03	4.19 ± 2.78
**Hydroxy-isocromans**	1-phenyl-6,7-dihydroxy-isochroman	-	-	-
	1-(3′-methoxy-4′hydroxy)-6,7-dihydroxy-isochroman	-	-	-
**Polyphenols, total**		198.0 ± 14.85	206.73 ± 150.08	551.42 ± 235.02

Source: Adapted from Cicerale et al., [81] and Rothwell et al., [93,94]. * Fresh weight. ROO: refined olive oil; EVOO: extra virgin olive oil.

**Table 2:** Clinical trials that evaluated the effect of different concentrations of olive oil phenolic compounds on inflammatory markers in patients with cardiovascular risk and CAD.

Reference	Population	Sample Size	Design	Duration	Intervention Group	Control Group	Outcomes
Visioli [127]	Patients with mild dyslipidemia	22	RCCT	2 × 7 weeks run-in: 3 weeks—ROO washout: 4 weeks—ROO	40 mL EVOO: 166 mg/L total HT	40 mL ROO: 2 mg/L total HT	↓ 20% TXB2 → Urinary excretion (24 h) of F2-isoprostanes (8-iso-PGF2α)
Pacheco [150]	Healthy subjects and patients with hypertriglyceridemia	28	RCCT	post-prandial (8 h) high-fat meal (72%) and 50 g noodles washout: 1 week run-in: 1 week—diet NCEP I + EVOO or ROO	50 g/kg² body surface area EVOO: 1125 mg/kg total polyphenols and 350 mg/kg tocopherols	50 g/kg² body surface area ROO: without polyphenols or tocopherols	Healthy and hypertriglyceridemia: smaller increment of the area under the sVCAM-1 and sICAM-1 curve
Fitó [18]	Patients with stable CAD	28	RCCT	2 × 3 weeks washout: 2 weeks—ROO	50 mL VOO: 161 mg/kg total phenolic compounds	50 mL ROO: 14.67 mg/kg total phenolic compounds	↓IL-6, ↓CRP, → sVCAM-1, →sICAM-1
Damasceno [144]	Patients with moderate hypercholesterolemia, without drug therapy or hormone replacement	18	RCCT	3 × 4 weeks run-in: 4 weeks MeDiet without washout among interventions	35–50 g MeDiet + VOO: 343 mg/kg total polyphenols	40–65 g MeDiet + walnuts: 13 mg/kg total polyphenols 50–75 g MeDiet + almonds: 11 mg/kg total polyphenols	→ CRP, → sVCAM-1, →sICAM-1
Moreno-Luna [19]	Women at stage 1 of essential hypertension or normal-high BP	24	RCCT	2 × 8 weeks run-in: 16 weeks—MeDiet washout: 4 weeks—MeDiet	60 mL MeDiet + VOO: 564 mg/kg–30 mg/day total polyphenols	60 mL MeDiet + ROO: without polyphenols	↓ CRP ↓ ADMA
Perez-Herrer [149]	Obese subjects	20	RCCT	post-prandial (2 and 4 h)—breakfast containing milk and muffins (made with different types of oils)	0.45 mL of oil/kg of body weight VOO: 400 mg/kg antioxidant phenols	0.45 mL of oil/kg of body weight SFO SOD: sunflower oil/canola oil + 2 mg of dimethylpolysiloxane SOP: sunflower oil/canola oil + 400 mg/kg VOO-phenol compounds extracted from the residue of olive oil production alperujo	VOO and SOP vs. SFO: ↓ NF-kB activation, ↑ protein level IkB-α, ↓ plasma LPS concentration → Levels of subunit mRNAs and NF-kB activators (p65, IKKβ, IKKα), inflammatory molecules (TNF-α, IL-1β, IL-6, MIF, c-Jun N-terminal kinase)
Widmer [141]	Patients with early atherosclerosis (endothelial dysfunction)	82	Paralell RCT	16 weeks usual diet	30 mL VOO + EGCG: 600 mg/kg total polyphenols	30 mL VOO: 340 mg/kg total polyphenols	VOO + EGCG vs. VOO: no differences OO (VOO and VOO + EGCG): ↓ sICAM-1, ↓ monocytes, ↓ lymphocytes, ↓ platelets, ↓ leukocytes ↑ plasma 8-isoprostanes
Camargo [148]	Patients with metabolic syndrome and no drug treatment	49	RCCT	post-prandial (4 h)—breakfast containing white bread and VOO Washout: 6 weeks—low-fat, high-carbohydrate diet	40 mL VOO with concentrations of phenolic compounds: High: 398 mg/kg Intermediate: 149 mg/kg Low: 70 mg/kg		High: → LPS, inhibited NF-κB, → serum levels IL-6, → TLR4 protein High vs. Low/intermediate: ↓ expression IL-6 High vs. Intermediate: ↓ IL-1β expression Low: ↑ NF- kB p65 subunit, ↑ serum IL-6 levels; ↑ TLR4 protein, ↑ LPS Low vs. High: 4 h postprandial, higher levels of LPS ↑ expression of TNF-α independent phenolic content
Santangelo [145]	Overweight and type 2 diabetes mellitus patients without insulin therapy	11	RCCT	2 × 4 weeks usual diet	25 mL EVOO: 577 mg/kg total phenolic compounds	25 mL ROO: without phenolic compounds	→ high-sensitive CRP, → IL-6, → TNF-α

**↑**: increase; → maintenance or no effect; ↓: decrease; RCT: randomized clinical trial; RCCT: randomized crossover clinical trials; OO: olive oil; ROO: refined olive oil; VOO: virgin olive oil; EVOO: extra virgin olive oil; HT: hydroxytyrosol; TXB2: thromboxane B2; NCEP: National Cholesterol Education Program; sICAM-1: soluble intercellular adhesion molecule-1; sVCAM-1: soluble vascular cell adhesion molecule-1; CAD: coronary artery disease; IL-6: interleukin-6; CRP: C-reactive protein; ADMA: asymmetric dimethylarginine; NF-κB: nuclear factor kappa B; SFO: sunflower oil; IκB-α: alpha inhibitor of NF-κB; mRNA: messenger ribonucleic acid; MIF: inhibitory factor of macrophage migration; MeDiet: Mediterranean diet; BP: blood pressure; EGCG: epigallocatechin 3-gallate; LPS: lipopolysaccharide; TLR4: toll-like receptor 4; TNF-α: tumor necrosis factor-alpha.

## Abbreviations

AA	arachidonic acid
CAD	coronary artery disease
COX	cyclooxygenase
CRP	C-reactive protein
EVOO	extra virgin olive oil
HDL	high density lipoprotein
HT	hydroxytyrosol
ICAM-1	intercellular adhesion molecule-1
IL	interleukin
INF-γ	interferon gamma
LDL	low density lipoprotein
LPS	lipopolysaccharides
LT	leukotriene
MCP-1	chemotactic monocyte protein
M-CSF	macrophage colony stimulating factor
MeDiet	mediterranean diet
MIF	macrophage migration inhibitory factor
MMP	matrix metalloproteinase
mRNA	messenger ribonucleic acid
NCEP	National Cholesterol Education Program
NF-κB	nuclear factor kappa B
NO	nitric oxide
NSAIDs	non-steroidal anti-inflammatory drugs
PG	prostaglandins
RCT	randomized clinical trial
ROO	refined olive oil
SFO	sunflower oil
sICAM-1	soluble intercellular-1-type adhesion molecule
SOD	mixture of oils (sunflower oil + canola oil)
sVCAM-1	soluble vascular cell adhesion molecule-1
Th	T helper cells
TLR	toll-like receptor
TNF-α	tumor necrosis factor-alpha
TX	tromboxanes
VCAM-1	vascular cell adhesion molecule-1
VOO	virgin olive oil
